# Acute Abscess of Teeth

**Published:** 1896-07

**Authors:** M. L. Rhein

**Affiliations:** New York.


ARTICLE VII.

### ACUTE ABSCESS OF TEETH.

DR. M. L. RHEIN, NEW YORK.

Presuming that the causes and symptoms of this form of the disease are familiar to you all, I desire to call your attention especially to the necessity for the most careful and thorough treatment pursued under the strictest antiseptic conditions, if it is to be hoped that a radical cure will be accomplished. If these precautions are followed with any ordinary common-sense treatment, a cure is readily effected. It is, however, unfortunately true that the graver form of this trouble—chronic alveolar abscess—results more frequently from a lack of attention to these precautions, namely, from improper dental work, than from any other source. A large portion of the treatment is necessarily the same, whether we have an acute stage of the disease or the chronic form.

It frequently falls to the lot of the dental practitioner to be placed in the happy position of being able to prevent an acute attack of alveolar abscess, and I shall therefore first take up your attention with this phase of the subject.

One of the errors commonly made in this disease is to delay operative treatment till pus is thoroughly formed and an external fistula is obtained, where frequently prompt antiseptic and surgical measures would prevent the threatened attack. With our present knowledge of the value of the more powerful germ-killing remedies, there remains no excuse for any delay in opening into the pulp-chamber of any tooth at any stage of this disease, or of a threatened attack of the disease.

The first step in the treatment of a tooth threatened with an alveolar abscess—and I may as well say in one where the disease exists in any stage—is the adjustment of the rubber dam over the diseased tooth, to preclude the possibility of the entrance of any of the germs in the oral secretions into the pulp-chamber. This should be the invariable rule.

The opening into the pulp-chamber should always be made on a direct line (or as close to that as possible) with the end of the root in single-rooted teeth, or on a line as far as possible commanding the ends of multi-rooted teeth. Do not fall into the error of utilizing an already established proximal cavity for this purpose. Many teeth have been ruined in this way, because the operators for the time being, thought they would save themselves the trouble of cutting through solid enamel. Do not depend on a small opening, but make it so large that there will be no question of an unobstructed view of the root-canals. It is far safer to remove a large quantity of healthy tooth-structure which can be perfectly restored by suitable filling-material, than to be compelled to operate in root-canals entirely by the sense of touch. The openings of the canals themselves should be enlarged by the use of suitable drills. The contents of the canals should then be thoroughly removed and by the careful performance of this portion of the operation is determined the successful prevention of the onsetting attack, or the cure of the disease, if it already exists.

As to the method of removing pulp-tissue in root-canals, it would be folly to lay down any one individual form of practice. The judgment of each operator must guide him in the choice of the best method for each individual case. There is no occasion for dilating on the necessity of a thorough removal of the pulp-tissue and the hermetical sealing of the ends of the roots, as well as of the canals themselves. Many cases of what, within a few hours, would result in an attack of acute abscess, present themselves to us before any purulent matter has formed. The patients are simply suffering from the intense pain that generally accompanies the oncoming of such an attack, and prompt antiseptic treatment, undertaken at an early stage, will generally abort the attack. The treatment pursued is the same as if pus had already formed.—*Cosmos.*

